# Editorial: Multi-organ linkage pathophysiology and therapy for NAFLD and NASH

**DOI:** 10.3389/fendo.2024.1418066

**Published:** 2024-06-05

**Authors:** Takefumi Kimura, Tomoo Yamazaki, Gabriel Rufino Estrela

**Affiliations:** ^1^ Department of Medicine, Division of Gastroenterology and Hepatology, Shinshu University School of Medicine, Matsumoto, Japan; ^2^ Consultation Center for Liver Diseases, Shinshu University Hospital, Matsumoto, Japan; ^3^ Department of Medicine, University of California, San Diego, La Jolla, CA, United States; ^4^ Department of Neurology and Neurosurgery, Discipline of Neuroscience, Federal University of Sao Paulo, Sao Paulo, Brazil

**Keywords:** non alcoholic fatty liver disease (NAFLD), non-alcoholic steatohepatitis (NASH), metabolic dysfunction-associated steatotic liver disease (MASLD), metabolic dysfunction-associated steatohepatitis (MASH), multi-organ linkage

## Introduction

Non-alcoholic fatty liver disease (NAFLD) and non-alcoholic steatohepatitis (NASH), now referred to as metabolic dysfunction-associated steatotic liver disease (MASLD) and metabolic dysfunction-associated steatohepatitis (MASH), respectively, are major public health problems associated with obesity and diabetes (1, 2). Their complex pathophysiology involves multiple organs and metabolic pathways, challenging diagnosis, risk assessment, and treatment. This Research Topic, “*Multi-organ linkage pathophysiology and therapy for NAFLD and NASH*”, provides new perspectives on organ linkages, pathogenesis, and therapeutic strategies for the management of NAFLD/MASLD and NASH/MASH.

## Extrahepatic malignancy risk

Previous studies have examined extrahepatic malignancy risk in NAFLD (3, 4), but often overlooked the influence of obesity on cancer risk. To address this gap, Albhaisi et al. matched NAFLD patients with a non-NAFLD group to minimize confounding and found no increased extrahepatic cancer risk in NAFLD. However, given the robust negative impact of obesity on carcinogenesis and the strong association between NAFLD and obesity, carcinogenesis in NAFLD warrants some attention in general practice, as previous studies have shown.

## Algorithm for assessing liver fibrosis risk in non-obese MAFLD

Asians, due to their genetic background, may encounter unique circumstances regarding the prognosis of non-obese (lean) NAFLD (5). Lee et al., researchers in Hong Kong, have proposed a distinctive sequential algorithm to assess liver fibrosis risk in non-diabetic overweight/obese individuals with metabolic dysfunction-related fatty liver disease (MAFLD). This algorithm integrates aspartate aminotransferase (AST) abnormalities and HOMA-IR≥2.5, alongside elastography, to stratify liver fibrosis risk in this population. Similar AST levels and liver fibrosis associations have been noted in other Asian cohorts, supporting these findings (6).

## Predicting fibrosis stage undergoing bariatric surgery

Bariatric surgery is emerging as a beneficial treatment for NAFLD/NASH. Huang et al. studied 373 patients who underwent intraoperative liver biopsy during bariatric surgery in China. They aimed to predict fibrosis stage F2 or higher (9.1%) using noninvasive models. In multivariate analysis, age, diabetes, c-peptide, and AST were significant predictors. Models like APRI, FIB-4, and HFS showed predictive accuracies (AUC: 0.745–0.781). These predictive abilities are expected to improve combined with MRI/US elastography and previously reported markers (7, 8).

## Comparative efficacy of GLP-1 receptor agonists in NAFLD

Novel therapeutics targeting G protein-coupled receptors are in development for obesity and diabetes (9). Glucagon-like peptide-1 receptor agonists (GLP-1RAs) are increasingly used for managing obesity and diabetes mellitus, necessitating an understanding of how different GLP-1RA formulations impact outcomes. Yuan et al. conducted a network meta-analysis of 14 randomized controlled trials, finding that twice-daily exenatide was most effective in reducing liver fat content, while once-daily semaglutide showed superior efficacy in reducing AST and ALT levels.

## Folate levels and NAFLD risk in adolescents

Folic acid deficiency heightens NAFLD risk in adults (10). Wen et al. used NHANES to study folate levels and NAFLD in adolescents (12–19 years). They found serum total folate or 5-methyl-tetrahydrofolate negatively correlated with CAP or liver stiffness. Mechanisms explored included inhibited lipid metabolism, impaired lipid transport, and folate-induced reductions in blood glucose and lipid concentrations.

## The liver-brain axis

Mai and Mao conducted a study investigating the causal relationship between NAFLD and cortical structure. They used Mendelian randomization methodology, incorporating genetic predictors of NAFLD and liver adiposity, alongside summary statistics from the ENIGMA Consortium’s genome-wide association study (GWAS). The findings revealed associations between NAFLD and liver adiposity with decreased surface area of the parahippocampal gyrus and increased thickness of the entorhinal cortex. These results suggest that NAFLD is linked to structural alterations in specific brain regions, emphasizing the potential influence of the hepatic-brain axis.

## The liver-bone axis

In a review by Chondrogianni et al., the link between NAFLD and osteoporosis was explored through experimental and clinical evidence. Both diseases are prevalent globally, often coexisting. Emerging data suggest common molecular pathways like sarcopenia, the RANKL-OPG-RANK pathway, and the Wnt pathway (11). However, not all epidemiological studies confirm a direct association. Comprehensive understanding of the liver-bone axis requires large prospective cohort studies and intervention trials supported by robust basic research.

## Closing remarks

This Research Topic incorporates a variety of research articles utilizing database studies, a valuable method for examining numerous cases and outcomes. However, we also stress the importance of cohort studies for the certainty of NAFLD diagnosis and detailed presentation of individual cases, supported by liver biopsy tissue diagnosis. By uncovering key mechanisms and identifying novel therapeutic targets, these studies will aid in developing personalized approaches for managing NAFLD/MASLD and NASH/MASH.

## Author contributions

TK: Writing – review & editing, Writing – original draft. TY: Writing – review & editing. GE: Writing – review & editing.
